# Factors impeding the supply of over-the-counter medications according to evidence-based practice: A mixed-methods study

**DOI:** 10.1371/journal.pone.0240913

**Published:** 2020-11-19

**Authors:** Nouf Aloudah, Areej Alhumsi, Nada Alobeid, Nourah Aboheimed, Hind Aboheimed, Ghada Aboheimed

**Affiliations:** 1 Clinical Pharmacy Department, King Saud University, Riyadh, Saudi Arabia; 2 Sales and Clinical Specialist Oncology, Becton Dickinson, Riyadh, Saudi Arabia; 3 Benefit Risk Assessment Department, Saudi Food and Drug Authority, Riyadh, Saudi Arabia; 4 Pharmacy Practice Department, Princess Nourah bint Abdulrahman University, Riyadh, Saudi Arabia; 5 Pharmaceutical Care Services, King Abdulaziz Medical City-Riyadh, National Guard Health Affairs, Riyadh, Saudi Arabia; The University of Sydney School of Pharmacy, AUSTRALIA

## Abstract

**Objective:**

Despite the positive attitudes pharmacists have toward evidence-based practices (EBPs), its application in community pharmacies in Saudi Arabia is lacking. Therefore, this study aimed to explore and assess EBPs by community pharmacists in Saudi Arabia when they dispense over-the-counter (OTC) medications for three minor ailments: diarrhea, cough, and the common cold.

**Research design and methods:**

We used a mixed-methods approach consisting of two study parts. The first was a quantitative investigation that used mystery shoppers. Four researchers, posing as mystery shoppers, visited 214 randomly selected pharmacies in the Riyadh region of Saudi Arabia. They used 14 questions from a standardized checklist to examine EBPs by community pharmacists. The qualitative part of the study entailed three focus-group discussions with 13 pharmacists from different community practice settings and explored factors that affected the application of EBPs when supplying OTC medications from the pharmacists’ point of view.

**Results:**

The analysis indicated that 40% of pharmacists dispensed OTC medications according to EBPs. Logistic regression analysis showed that one question, "Describe your symptoms", predicted the correct supply of OTC medications (p = 0.021). The qualitative section of the study identified nine factors that affected EBP. Some of these factors facilitated EBP, such as established patient-pharmacist relationships, some acted as barriers such as conflicts between available evidence, while other factors could either facilitate or hinder EBPs, such as the health literacy of the patient.

**Conclusion:**

Given that dispensing OTC medication is a core function of pharmacists, this study uncovered low adherence to EBPs by community pharmacists in Saudi Arabia when dispensing OTC medication for three minor ailments: diarrhea, cough, and the common cold. Furthermore, this study identified a number of explanatory factors for this low adherence. Targeting these factors could help change the behavior of pharmacists and decrease undesirable outcomes.

## Introduction

Providing medication without a prescription is one of the core functions of community pharmacies [1]. There are more than 300,000 over-the-counter (OTC) medications in the United States market that fall under more than 80 therapeutic classes [1]. In Saudi Arabia, there are more than 9,000 OTC medications registered by the Saudi Food and Drug Authority [2]. While OTC medications reduce the cost of healthcare [1], medication safety mandates that the pharmacist assist patients with choosing an appropriate OTC medication.

Studies investigating why pharmacists do not supply OTC medications according to evidence-based practices (EBPs) have identified a number of influencing factors. First, OTC medications sold in community pharmacies may be based on commercial gain [3]. In addition, pharmacists may not be familiar with how to access EBP information, there may be limited available evidence regarding medication effectiveness, and challenging patients’ requests [4]. Pharmacists play a crucial role in ensuring recommendations are guided by rational EBPs. One previous study has shown that community pharmacists and pharmacy assistants recognized that consumers lack knowledge regarding OTC medication risks and, thus, there was a need to acquire consultation skills [5]. Yet, consumers were not aware of the professional role of pharmacists [5]. High quality OTC consultations are particularly vital in the face of global deregulation of prescription-only medications and the potential for self-selection of medications by patients [6]. For example, one study conducted in Saudi Arabia found that more than 35% of medications were self-selected by patients, of which 50% were prescription-only [7]. Moreover, 45% of the participants did not disclose any prescribed medication being used at home [7]. Therefore, as pharmacists are the most easily accessible healthcare professionals, there is need for pharmacists to use clinical-trial evidence that follows the principles of EBPs.

While several recent studies have reported that community pharmacists held positive attitudes towards EBPs [6, 8], these beliefs were not always put into practice. A study exploring the appropriateness of self-management cases with OTC medications in Scotland using a mystery-shopper approach showed that 96% of the visits were conducted without any consultation. Furthermore, the study identified a lack of pharmacy-specific quality standards [9]. However, to our knowledge, no study has used a mixed-method approach to assess and explore the point of view of community pharmacists in Saudi Arabia. Therefore, we aimed to evaluate the level of EBP adherence by pharmacists and explore the factors underlying the use of EBP when dispensing OTC medications for diarrhea, cough, and the common cold by community pharmacists using a mixed-methods approach.

## Research design and methods

This study used a mixed-methods approach that consisted of two study arms: quantitative and qualitative [10]. The quantitative arm used a mystery-shopper method for assessing the use of EBP in community pharmacies that were randomly selected from the Riyadh region of Saudi Arabia. The mystery shoppers acted as patients with one of three developed scenarios for cough, diarrhea, or the common cold. The qualitative study arm consisted of several focus-group (FG) meetings with a sample of community pharmacists working in Riyadh.

### The mystery-shopper method

Four researchers posed as mystery shoppers (N.O, A.H., H.A., N.A.B) and used one of three scenarios when visiting the pharmacies. Three minor ailments were chosen based on the following criteria: they were sufficiently common to generate cases during the data collection period, there was available literature regarding evidence for their management, and the ease of diagnosis by the pharmacist from the patients' descriptions of their symptoms.

The Quest/Scholar MAC Checklist was used as a guide for the development of the scenarios. Quest/scholar MAC is an abbreviation mnemonic for two sets of elements. First, the (Qu) and (Scholar MAC) represent: **Qu**ickly and accurately assess the patient by asking about the following: **S**ymptoms, **C**haracteristics, **H**istory, **O**nset, **L**ocation, **A**ggravating factors, **R**emitting factors, **M**edication use (including prescription and nonprescription products), **A**llergic reactions, **C**oexisting conditions; and the abbreviation (Est), which represents the following actions: **E**stablish that the patient can use a self-care product, **S**uggest an appropriate self-care product, **T**alk with the patient about the product [11]. Quest/Scholar MAC is a checklist that helps clinicians ask a series of questions to assess a patient’s condition and select the most appropriate OTC medication. It was developed to standardize and improve how patients with self-care issues are treated [11].

To assess the accuracy of supplying OTC medication that aligned with EBPs, a literature review was conducted to identify recent evidence for treating the three minor ailments (Table 1). Mystery shoppers received two training sessions. The first occurred before a pilot study and included role playing and working through possible pharmacy scenarios according to what might happen. Mystery shoppers were given explicit instructions on how to conduct the pharmacy phase of the study, especially in regards to not providing information unless requested by the pharmacist. The second session occurred after the pilot study and provided additional instructions regarding buying products if required. Each researcher was assigned 80 pharmacies to be visited using a list obtained from the Saudi Ministry of Health. Immediately following the visit, the researcher filled out a checklist (audio recordings of the visits were not authorized). No consent was provided by the pharmacist or pharmacy as obtaining it could have biased their responses. Periodically, a decision to buy products occurred, so as to not affect the pharmacists’ reactions.

**Table 1:** Scenarios used in the mystery-shopper approach.

Scenario 1	Scenario 2	Scenario 3
The mystery shopper enters the pharmacy and says, "I have nasal congestion.” If asked, the mystery shopper tells the pharmacy staff that:	The mystery shopper enters the pharmacy and says, "I have diarrhea." If asked, the mystery shopper tells the pharmacy staff that:	The mystery shopper enters the pharmacy and says, "I have a cough." If asked, the mystery shopper tells the pharmacy staff:
She is 22 years old, female, not pregnant and not a smoker. She is the only one in her family who had caught the flu. She has a scratchy throat and cannot breathe out of her nose because it is very congested. She has been experiencing a sore throat for two days and nasal congestion for one day.	She is 22 years old, female, not pregnant and not a smoker. She had not traveled for one year but had eaten fast food the day before. She is the only one in her family with diarrhea. She had vomited once and has stomach cramping and thirst with no fever. Her diarrhea is watery with no blood or mucus, which started one day previously and occurs 2–3 times a day.	She is 22 years old, female, not pregnant and not a smoker. She is the only one in her family who has a cough. The cough is dry with no mucus and not associated with blood. The cough started three days previously.
		She does not have other conditions or takes other medications.
She has no other conditions or takes other medications.		
	She does not have other conditions or takes other medications.	
**EBP Recommendations**	**EBP recommendations**	**EBP recommendations**
Oral and intranasal decongestants, antihistamines and analgesics.	Loperamide or bismuth subsalicylate	Antitussives or expectorants,
		dextromethorphan or guaifenesin, or pelargonium sidoides root extract
Comfort measures: use of menthol sweets or steam inhalation		

### Data collection

The scenarios and the mystery-shopper approach were piloted with nine community pharmacies to ensure consistency. Modifications of the checklist were undertaken when needed (for instance, adding the appropriate OTC medication to the scenario to facilitate assessment by the mystery shopper). Mystery-shopper visits were conducted between December 2016 and January 2017.

### Sample size and statistical analysis

The estimated sample size for the mystery-shopper method was calculated to be 207 pharmacies. The target population was drawn from pharmacies in Riyadh, Saudi Arabia. Riyadh has more than 2,366 pharmacies in the private sector, which include community pharmacies and those incorporated in private hospitals. The sample size was determined using the followed formula:

$$n=\frac{z^{2}P(1-P)}{d^{2}}$$

In this formula, *Z* is the statistic corresponding to a 95% confidence level, which was 1.96. *P* is the prevalence (17%) [12], and *d* is the accuracy level of the precision, which was 5%. Statistical analyses were performed using the Statistical Package for the Social Sciences version 21 (IBM Corp., Armonk, NY, USA). A P-value ≤ 0.05 indicated statistical significance. Odds ratios (ORs) and 95% confidence intervals (CIs) were calculated using multivariable logistic regression analyses. The outcome (dependent variable) was “OTC prescription according to EBP,” and the predictors (independent variables) were the 14 items of the checklist, in addition to the presenting symptoms. All variables were included in the model. Model fitness was tested using the Hosmer-Lemshaw test (p = 0.423).

### The FG discussions

Community pharmacists from large chains and individual pharmacies were recruited for discussions. A topic guide was used during the focus groups (see S1 File). The questions were based on the Theoretical Domain Framework (TDF) [13]. TDF is an integrative framework of 33 psychological theories that aims to understand behavior change. The TDF consisted of 14 domain underpinning each theory. The used questions in the topic guide were derived from the questions of a consensus study by Michie [14] and modified to reflect Saudi culture and community pharmacist behavior towards supplying OTC medications according to EBPs. Previous responses by the participants and levels of interest guided the flow of questions. The FG discussions were recorded and transcribed verbatim using Atlas.ti software (Scientific Software Development GmbH, Berlin, Germany) by the researchers (N.O. and H.A.). Moderators took notes during the FGs and wrote a summary following each discussion. The FG discussions were conducted at King Saud University, Pharmacy College and took approximately 60 to 90 minutes each.

### Data analysis

FG transcripts were checked against the audio recordings and anonymized. All transcripts and audio-recordings were securely kept in password-protected computers. Thematic content analysis was undertaken to analyze the data. This process involved systematically analyzing the transcripts, assigning codes to ideas, and gathering examples of those codes from the text. Several cycles of coding were required to reach the final version of the coding manual (see S1 Table. Focus Group demographic characteristics and final coding manual).

### Ethical approval

Ethical approval for the research project was obtained from the King Saud University Institutional Review Board, College of Medicine, on May 26, 2016 (reference number 16/0344/IRB).

## Results

Two hundred and fourteen pharmacies from five different districts in Riyadh, Saudi Arabia were visited by the mystery shoppers. Table 2 lists the number of pharmacies visited in each district and the frequency of each scenario used. All responses were included in the analysis. Thirteen pharmacists participated in the FG discussions. The pharmacists' ages ranged from 24–41 years. All of the pharmacists were male, non-Saudis and their practice experience varied from 2–16 years. Content analysis took place after each FG. Following the third FG no new themes emerged and saturation had been attained. A description of the sample is presented in S1 Table (Focus Group demographic characteristics and final coding manual).

**Table 2:** Percentages of pharmacies visited in each district of Riyadh City and the frequency of the three scenarios.

District	Number (%)
Eastern (Al-Rawda)	43 (20.1)
Northern (Al-Shamal)	45 (21.0)
Southern (Al-Shifa)	40 (18.7)
Western (Irqa)	45 (21.0)
Central (Olaya)	41 (19.2)
**Scenario**	
Common cold	69 (32.2)
Cough	74 (34.6)
Diarrhea	71 (33.2)

### Mystery shopper study results

Eighty-six (40.2%) of the community pharmacists supplied an OTC medication according to EBPs. Table 3 lists the checklist item responses (items 1–14) and an assessment of the OTC medications provided according to EBP (item 15).

**Table 3:** Results of the checklist items used to assess responses by pharmacists and the percentage who did not supply OTC medications according to EBPs.

Checklist Item	Response	Frequency (%)
1-Did the pharmacist ask about your age?	No	156 (72.9)
2-Did the pharmacist ask about your maternity status?	No	203 (94.9)
3-Did the pharmacist ask about your social history?	No	204 (95.3)
4-Did the pharmacist ask about your family history?	No	208 (97.2)
5-Did the pharmacist ask about your main and associated symptoms?	No	50 (23.4)
6-Did the pharmacist ask you to describe your symptoms?	No	95 (44.4)
7-Did the pharmacist ask you what has been done to relieve the symptoms and what has been done so far?	No	195 (91.1)
8-Did the pharmacist ask when the symptoms began?	No	148 (69.2)
9-Did the pharmacist ask about the frequency of symptoms (if applicable)?	No	198 (92.5)
10-Did the pharmacist ask what made the symptoms worse?	No	206 (96.3)
11-Did the pharmacist asked what made the symptoms better?	No	209 (97.7)
12-Did the pharmacist ask about previous medications?	No	205 (95.8)
13-Did the pharmacist ask about allergies?	No	210 (98.1)
14-Did the pharmacist ask about other conditions?	No	199 (93)
**15-Did the pharmacist supply an OTC drug according to EBP?**	**No**	**128 (59.8)**

The multivariable logistic analysis revealed that item 6 was a significant determinant of EBP prescription (OR: 2.22; 95% CI (1.13–4.41); p = 0.021), in addition to the symptoms of the clinical scenario (p = 0.001). Patients who had a common cold and cough were more likely to get OTC medication according to EBPs (OR: 5.36; 95% CI (2.46–11.7); p< 0.001 and OR: 5.19; 95% CI (2.4–11.21); p< 0.001, respectively) when compared to patients with diarrhea (Table 4). The estimated odds ratio was nearly two times greater when the pharmacist asked the shoppers to describe their symptoms.

**Table 4:** Relationship between checklist questions and the provision of OTC medications according to EBPs.

Checklist questions	Odds ratio	p-value	95% Confidence interval
1- Did the pharmacist asked about your age?	0.937	0.859	0.458–1.918
2- Did the pharmacist asked about your maternity status?	2.334	0.258	0.538–10.127
3- Did the pharmacist asked about your social history?	0.999	.	.
4- Did the pharmacist asked about your family history?	1.397	0.726	0.216–9.047
5- Did the pharmacist asked about the main and associated symptoms?	0.621	0.217	0.292–1.323
6- Did the pharmacist asked you to describe your symptoms?	2.233	0.021	1.131–4.407
7- Did the pharmacist asked you what has been done to relieve the symptoms and what has been done so far?	0.815	0.766	0.213–3.124
8- Did the pharmacist asked when the symptoms began?	0.541	0.130	0.244–1.198
9- Did the pharmacist ask about the frequency of the symptoms (if applicable)?	1.810	0.401	0.453–7.235
10- Did the pharmacist ask what makes the symptoms worse?	6.198	0.125	0.604–63.602
11- Did the pharmacist asked what makes the symptoms better?	0.073	0.111	0.003–1.827
12-Did the pharmacist ask about previous medication?	0.819	0.841	0.117–5.751
13- Did the pharmacist asked about allergies?	3.789	0.266	0.363–39.532
14- Did the pharmacist asked about other conditions?	1.179	0.810	0.308–4.510
**Presenting symptoms**			
Common cold vs. diarrhea	5.36	< 0.001	2.46–11.7
Cough vs. diarrhea	5.19	< 0.001	2.4–11.21

“r-squared 0.115, Chi-square 31.986, p = 0.004”.

### FG discussions

The content analysis of the community pharmacists' point of view towards supplying OTC medications according to EBP identified nine factors that influenced their behavior. These factors were summarized under three main domains: the system, the pharmacist, and the patient.

### The system

#### Conflict and/or lack of EBP guidelines for OTC medications

A lack of OTC-medication evidence-based treatment guidelines for some ailments limited application of EBPs by pharmacists. Furthermore, there was a reported lack of national guidelines and, instead, the pharmacists needed to explore and choose from several international guidelines. As one pharmacist stated: "*…*. *there are American*, *Australian*, *and British guidelines… and not every pharmacist has enough knowledge to choose from them [to answer their queries]" (2–4)*. Moreover, keeping current with changes in guidelines, as well as conflicts between old and new guidelines, was also described by a pharmacist as problematic: "*The other thing is the evidence-based medicine consistently changes; maybe this year it is correct according to evidence-based and next year its not…" (3–1)*.

#### Time restrictions and a focus on sales by pharmacy management

There is limited time available for pharmacist-patient communication. For instance, one pharmacist stated that: *"There is not enough time because there are other requirements*. *The patient expects minimum information*, *and the time we have does not give us the space to provide each patient with the information he needs*. *… I cannot even say hello to them*.*" (1–1)*. Sales also played a role, as one pharmacist stated: *"If you want me to supply [medication] according to evidence-base*, *do not obligate me with sales*.*" (1–1)*. Another pharmacist said: *"Honestly*, *he [the manager] knows that you may not adhere to the guidelines*, *but he turns a blind eye because he sees a benefit from another aspect [i*.*e*., *sales]" (1–4)*.

#### A need for checklists and/or reminders to facilitate pharmacist adherence

A need for checklists for the evidence-based supply of OTC medications to facilitate pharmacist adherence was reported. As one pharmacist stated: *"…*.*if there is a checklist that we rely on*…*" (3–1)*, and another pharmacist said: *"First of all*, *I need a reminder to remind me to prescribe according to evidence-base until I get used to it*.*” (1–1)*.

#### Pharmacy location

The influence of and/or conflict between pharmacists reportedly affected their behavior. In one instance, a pharmacist stated: "*…the patient said why did you refused to dispense to me while your colleague [the nearby pharmacist] did*?*"* (*2–1*). The effect of whether a pharmacy location was adjacent to the physician's location was also identified as an important factor in influencing the supply of OTC medication according to EBPs. For example, one pharmacist stated: *"Communication with physicians would be more appropriate to prescribe medications according to EBP*. *So*, *if a patient comes with ambiguous prescriptions*, *I refer the patient back to him*.*" (1–3)*.

### The pharmacist

#### Pharmacists’ knowledge of medication

Pharmacists’ knowledge of medications (pharmaceutical ingredients) was reported as a reason for not following guidelines. For instance, one pharmacist said: *"If I know the composition of the medication*, *and there is no contraindication to prescribe this medication to a patient six years old or younger*, *I should provide it to the public if there will not be a medical mistake*.*" (1–2)*. Pressure was also placed on pharmacists to supply brand name medications rather than generic therapeutic substitutes. According to one pharmacist’s opinion: *"If I am committed to using EBP then …*. *they should be treated based on the scientific name*, *not the brand name" (1–2)*.

#### Pharmacists' perceptions of their professional identity was empowered by giving several OTC medication options

One pharmacist believed that following EBP guidelines might negatively affect and further weaken the professional identity of pharmacists. He believed that the ability to provide patients with several options, not just EBP options, shaped professional identity. As the following pharmacist stated: *"Without counseling*, *there is no need for the pharmacist in the pharmacy…*.*” (1–2)*. Another pharmacist stated: *"It would be a boring and non-interesting process where I would be directly medicating without having a discussion with the patient*.*" (2–1)*. Furthermore, a lack of self-confidence and morality, i.e., focusing on pleasing managers, was perceived by some participants to be a barrier. As one pharmacist stated: *"There will be a difference between pharmacists due to different experiences*, *training*, *and the morality of the pharmacists*. *If they were dealing with patients as a healthcare provider or directed by something else… It all depends on the conscience of the pharmacist*…*" (2–2)*. Moreover, some pharmacists expressed negative emotions such as a fear of patient reactions: *"If I did or did not prescribe according to EBP [he meant according to patients’ desires and not his own judgment]*, *the patient will shout and overreact*.*” (1–1)*.

#### Incentives

Some participating pharmacists mentioned that incentives should be given to encourage dispensing OTC medications according to EBPs. For instance, one pharmacist stated that: *"… there should be financial and non-financial incentives that will encourage adherence*.*" (1–1*). Another participant mentioned the following possible incentive: ‘…*it can be…employee of the month*, *for example*, *an employee from each district to receive a certain honor’ (3–3)*.

### The patient

#### Patient health literacy and culture

A patient’s health literacy and culture also affected pharmacist behavior. One pharmacist stated: *"I depend on the patient’s level of education*, *so if the patient has a high level*, *I explain this medicine but not the opposite…" (1–1)*. Participants described a need to educate the community about the role of the pharmacist in providing correct, required, and individualized drug information. For example, a pharmacist stated that: *"The problem with the culture is that they [patients] do not appreciate the role of the pharmacist*, *and they think the pharmacist is just like a salesperson*. *Also*, *they do not know that you have enough knowledge*, *so this will create a gap and resistance between us*.*" (2–3)*. Some participants also reported conflicts between modern and herbal medicine when dispensing OTC medication according to EBPs. For instance, one pharmacist said, *"There was a patient who came to the pharmacy*, *and I dispensed an antibiotic*, *and he noted {I don't want antibiotics I read on internet Pomegranate peel may be helpful*.*} … (1–1)*.

#### Patient-pharmacist relationships

Patient-pharmacist relationships established through counseling, time, and good communication skills facilitated the supply of OTC medications according to EBPs. As one pharmacist stated: *"Communication with the patients can the smooth way where you can convince him by explaining what the consequences of the medication are*. *For example*: *if I advised a patient by explaining to him what the benefit of such medication is*.*" (1–3)*.

## Discussion

Using a mixed-method approach allowed us to assemble a comprehensive understanding of how pharmacists dispense OTC medications using EBPs in Saudi Arabia. However, other methodologies have been employed to assess how community pharmacists dispense OTC medication according to EBPs. For instance, Collins et al. [15] used more than 500 mystery-shopper visits to 36 community pharmacies in Australia. Their study showed that less than half of the visits (46%) did not provide OTC medications according to EBPs, only 54% of the pharmacists visited provided products according to guidelines. Although their results were slightly better than ours, compliance with EBPs was surprisingly low as the study intervention included repeat visits to the same pharmacies and provided feedback and coaching after each mystery-shopper visit. The Collin et al. study did show improved results by providing continuous coaching to community pharmacists. Although, education and training are not the only interventions needed to improve EBPs. A mixed-methods study conducted by Watson et al in Scotland, which consisted of 195 observed consultations followed by 95 post-consultations, showed that most OTC consultations were not compliant with the British professional and good practice guidelines [16]. Their study further illustrated that, unlike non-product consultations, product requests predominated and received low scores in regards to compliance with the guideline [16].

Investigating the factors that influence whether or not pharmacists supply OTC medications according to EBPs could help with devising and implementing operational interventions [17]. Halila et al. investigated these factors by conducting a cross-sectional study examining more than 8,000 community pharmacists in Brazil using a self-administered, anonymized survey aimed to assess the knowledge of EBP and important factors to consider when recommending an OTC medicine [18]. Their results showed that pharmacists with less experience were significantly more likely to consider customer preference as the most important factor. Moreover, more than 60% of the participants searched Google for answers to questions or problems compared to 50% who consulted books or guides. In addition, their study showed that most pharmacists did not know the meaning of terms related to EBP including meta-analysis, publication bias and p-value. The Halila et al. study identified gaps in pharmacists’ EBP-related knowledge, as well as issues pertaining to how trustworthy information was obtained [17]. Similarly, the quantitative arm of our study demonstrated that most participating community pharmacists did not gather the proper information needed to understand cases and adequately provide OTC medication according to EBPs.

In our study, asking a patient about their symptoms significantly predicted whether OTC medication was provided according to EBPs and appeared to play a key role in the communication between pharmacists and patients. Similarly, the Watson et al. study demonstrated that patients were more likely to exchange information if they asked about symptom treatment as opposed to asking for a product. Furthermore, that study showed that the patients did not engage in this approach due to concerns about their privacy [16]. The results of the mystery shopper arm of our study also confirmed the importance of good patient-pharmacist communication. These results indicate that it is important to explore those factors that contribute to communication failures. This was investigated in our second study arm, the in-depth FG discussions, which identified a number of systemic, pharmacist, and patient-related factors. Similarly, a qualitative study conducted with 26 community pharmacists in Northern Ireland explored the role of EBPs when dispensing products and showed that pharmacists believed product safety was the predominant factor that determined their decision (even if the medication lacked evidence of effectiveness) [19]. Another in-depth interview study with 16 pharmacists in England explored how pharmacists used evidence when supplying OTC medication and demonstrated that product selection was based on personal judgment and patient feedback rather than evidence [20]. The pharmacists in these studies acknowledged that they sold ineffective OTC medications if they were preferred by patients [19, 20].

Our study also illustrated the role of administration-related factors that can further restrict decisions made by pharmacists, such as a focus on sales by management. Moreover, the in-depth-interviews indicated that pharmacists desired incentives to engage in EBP adherence. Empowering pharmacists through corrective managerial actions could be undertaken by designing incentivized schemes that have previously been successfully applied to overcome poor performance [21].

Designing and implementing plans to improve the supply of OTC medication according to EBPs rather than blaming pharmacists for following patient preferences and providing ineffective medications is necessary. To further this goal, we used a systematic approach based on theory by using the TDF to build the topic guide to explore and understand how pharmacists engage in EBPs in Saudi Arabia. Strategically constructing interventions requires considering a full range of options and systematically evaluating them [22]. Such approach can help to sustain EBPs in community pharmacies in everyday practice [22].

### Limitations

Our dataset was limited by the available tools used to assess pharmacist behavior. While using mystery shoppers can overcome some of these limitations, their use has its limitations [23]. Blinding mystery-shopper visits was a concern and could have affected the validity had the pharmacists been aware of the visits [24]. Therefore, we took the approach of not informing the pharmacist of the study and count on the researcher answering the checklist as soon as the leave the pharmacy. Furthermore, the mystery shoppers did not have previous experience with mystery shopping. However, our training consisted of providing explicit information, and the shoppers undertook role-playing activities before the pilot study, which was followed by another session to manage any required changes to the process. Our study also used a standardized checklist to build the scenarios and assess the responses of the pharmacists to strengthen outcome validity. The external validity of our study was strengthened by the large number of visits conducted. The internal validity was confirmed as there were no significant differences in the number of OTC medications according to EBPs found between the researchers’ results (p = 0.89).

There are 7,322 community pharmacies and 12,506 pharmacists working in Saudi Arabia [25]. Our data was collected from only one region, and we expect to see differences in other regions of Saudi Arabia. Specifically, there is evidence that gender might influence interactions and patient satisfaction when receiving health care [26]. All community pharmacists in Saudi Arabia were males and most were non-Saudis. However, this situation changed after we had collected our data, as females were not allowed to work in community pharmacies until 2018 [27, 28]. The generalizability of our study may also have been limited as prescription medication is often sold from community pharmacies without prescriptions. Although the law states that prescription medication should be dispensed only on the presentation of a prescription, many community pharmacies do not follow this regulation [7, 29].

Our study was strengthened by using a theory-based approach when designing the qualitative arm. We explored the behavior of community pharmacists using the Theoretical Domain Framework, which helped to explicitly analyze the pharmacist's point of view. Moreover, the quantitative study arm uncovered numerous variables to target for interventions.

## Conclusion

Studying the supply of OTC medications according to EBPs using a mixed-method approach suggests that pharmacists are not at fault for taking a non-EBP approach. Given that providing OTC medication is a core function of pharmacists, using the factors underlying non-EBPs that were identified in this study to design interventions should help to change this behavior and decrease undesirable outcomes.

## Supporting information

S1 File Focus group topic guide. (DOCX)

S1 Table Focus group final coding manual. (DOCX)
