# Supplementary material for: Factors impeding the supply of over-the-counter medications according to evidence-based practice: A mixed-methods study
Source: PLoS One. 2020 Nov 19;15(11):e0240913. doi: 10.1371/journal.pone.0240913 (PMC7676695; doi:10.1371/journal.pone.0240913)
Supplement: S1 File — (DOCX) [file pone.0240913.s001.docx]

S1 File. Factors impeding the supply of over-the-counter medications according to evidence-based practice, 1/3/2017 NO, NA- **Focus Group Topic Guide**

I want to thank you for taking the time to meet with me today. I would like to talk to you about your experiences with the evidence-based supply of non-prescription medicines/ over-the-counter (OTC) in community pharmacies in Saudi Arabia, including what helps and hinders your actions. My focus will be mainly on the three major diseases we see: cough, common cold, and diarrhea. But during the conversation, it is OK if you talk about other conditions or cases.

The focus group should take less than 90 min. I will be recording the session because I do not want to miss out on any of your comments. I will be taking some notes during the session. All responses will be kept confidential. This means that your answers will only be shared with research team members, and we will ensure that any information we include in our report does not identify you as the respondent. Remember, you do not have to talk about anything you do not want to, and you may end the interview at any time.

If you can introduce yourself, and how many years of experience you have in the community pharmacy outside Saudi Arabia (SA) and inside SA.

I would like to start with some basic questions about what do you know about evidence-based medicine (EBM) for cough, common cold, and diarrhea when dispensing of OTC?

1. What do you think the EBM for cough, common cold, and diarrhea says when dispensing of OTC?
2. Is it obligated to use EBM when prescribing? I mean, do you know you should be using EBM when dispensing of OTC for cough, common cold, and diarrhea, or is it OK not to use it? Do you know why?
3. How easy or difficult do you find performing the use of EBM when dispensing of OTC for cough, common cold, and diarrhea to the required standard in the required context?
4. What is the purpose of the EBM when dispensing of OTC for the three diseases mentioned? (If they do not answer, it is OK not only these diseases but also the others?)
5. What do you think about the credibility of EBM source for cough, common cold, and diarrhea?
6. Is using EBM compatible or in conflict with professional standards/identity of the pharmacy? (Prompts: moral/ethical issues, limits to autonomy). Would this be true for all community pharmacists involved?
7. Do you remember any problems have you encountered when you used EBM for cough, common cold, and diarrhea when dispensing of OTC?
8. What would help when you are dispensing of OTC according to the EBM for cough, common cold, and diarrhea?
9. How confident are you that you can use EBM for cough, common cold, and diarrhea despite the difficulties you faced when dispensing of OTC?
10. What do you think will happen if you use EBM for cough, common cold, and diarrhea when dispensing of OTC? (prompt regarding themselves, patients, colleagues, and the organization; positive and negative, short term and long-term consequences)
11. What are the costs of using EBM, and what are the costs of the consequences of using EBM when dispensing of OTC for cough, common cold, and diarrhea?
12. When you dispense of OTC, what do you think will happen if you do not use EBM? (prompts)
13. Do you think the benefits of using EBM when dispensing of OTC for cough, common cold and diarrhea outweigh the costs?
14. How much do you want to use EBM for cough, common cold, and diarrhea?
15. How much do you feel you need to use EBM for cough, common cold, and diarrhea?
16. In practice, are there other things you want to do or achieve that might interfere with using EBM when dispensing of OTC for cough, common cold, and diarrhea?
17. What kind of incentives can motivate you to follow EBM when dispensing of OTC for cough, common cold, and diarrhea?
18. How much attention will you have to pay to use EBM for the three diseases?
19. In everyday dispensing, will you remember to use EBM? How?
20. Might you decide not to use EBM when dispensing of OTC for cough, common cold, and diarrhea? Why? (prompt: competing tasks, time constraints)
21. To what extent do physical or resource factors facilitate or hinder the use of EBM when dispensing of OTC for cough, common cold, and diarrhea? Prompts: are the necessary resources for cough, common cold, and diarrhea when dispensing of OTC available to you as community pharmacist?
22. To what extent do social influences facilitate or hinder the use of EBM for cough, common cold, and diarrhea when dispensing of OTC? (prompts: peers, managers, other professional groups, patients, relatives)
23. Will you observe others using EBM for cough, common cold, and diarrhea (i.e., have role models)?
24. To what extent do emotional factors facilitate or hinder the use of EBM for cough, common cold, and diarrhea when dispensing of OTC? How do emotions affect the use of it?
25. What preparatory steps are needed to use EBM when dispensing of OTC? (prompt: individual and organizational)
26. Are there procedures or ways of working that encourage use EBM for cough, common cold, and diarrhea when dispensing of OTC?
27. How well equipped/comfortable do you feel to use our EBM when dispensing OTC medication?
28. Who needs to do what differently when, where, how often, and with whom?
29. How do you know whether the behavior has happened?
30. Is the use of EBM for cough, common cold, and diarrhea when dispensing of OTC considered as a new behavior or an actual behavior that needs to become a habit?
31. How long are changes going to take? Are there systems for maintaining long-term changes?

That is all the questions I have for you; is there something vital that we have not covered that you would like to say?

Thank you!
