# Supplementary material for: Factors impeding the supply of over-the-counter medications according to evidence-based practice: A mixed-methods study
Source: PLoS One. 2020 Nov 19;15(11):e0240913. doi: 10.1371/journal.pone.0240913 (PMC7676695; doi:10.1371/journal.pone.0240913)
Supplement: S1 Table — (DOCX) [file pone.0240913.s002.docx]

S1 Table. Factors impeding the supply of over-the-counter medications according to evidence-based practice

Focus Groups demographic characteristics and final coding manual

Demographic data for pharmacist:

(First focus group) Date: 4-Jan-2017

| **pharmacist** | **Age** | **Nationality** | **Education level** | **Number of years of works** |
| --- | --- | --- | --- | --- |
| 1 | 26 | Egyptian | Bachelor’s degree | 2 years in Saudi and 2 years in Egypt |
| 2 | 27 | Egyptian | Bachelor’s degree | 1 year in Saudi and 1 year in Egypt |
| 3 | 31 | Egyptian | Bachelor’s degree +Clinical diploma | 5 years in Saudi and 5 years in Egypt |
| 4 | 24 | Egyptian | Bachelor’s degree | 1 month in Saudi and 2 years in Egypt |

(Second focus group) Date: 22-Jan-2017

| **pharmacist** | **Age** | **Nationality** | **Education level** | **Number of years of works** |
| --- | --- | --- | --- | --- |
| 1 | 31 | Egyptian | Bachelor’s degree | 9 years in Saudi and 7 months in Egypt |
| 2 | 33 | Egyptian | Bachelor’s degree | 8 years in Saudi and 2 years in Egypt |
| 3 | 40 | Egyptian | Bachelor’s degree | 15 years in Saudi |
| 4 | 26 | Egyptian | Bachelor’s degree +Master’s degree | 1 year in Saudi and 1 year and a half in Egypt |
| 5 | 42 | Egyptian | Bachelor’s degree | 10 years in Saudi and 3 years in Egypt |
| 6 | 24 | Egyptian | Bachelor’s degree | 3 years in Saudi and 7 years in Egypt |

(Third focus group) Date: 29-Jan-2017

| **pharmacist** | **Age** | **Nationality** | **Education level** | **Number of years of works** |
| --- | --- | --- | --- | --- |
| 1 | 24 | Egyptian | Bachelor’s degree | 2 years in Saudi and 1 years in Egypt |
| 2 | 41 | Egyptian | Bachelor’s degree | 8 year in Saudi and 5 years in Egypt |
| 3 | 36 | Egyptian | Bachelor’s degree | 5 years in Saudi and 4 years in Egypt |

| **Code/ factor** | **Comments and things mentioned by the participants** |
| --- | --- |
| 1. Pharmacist knowledge of medication   Pharmacist knowledge of medication (pharmaceutical ingredients) resulted in not following the guidelines | The conflict between guideline and new medications (old guideline) |
|  | The drug is not indicated for children less than 6 years, so the pharmacist didn’t convince with guideline and changed the dose of the drug |
| 1. Experience   Knowledge gained from experience | More expertise of pharmacist = more evidence-based prescribing |
|  | Knowledge of pharmacist |
|  | Pharmacist s experience |
|  | Background knowledge |
| 1. Searching the information   Knowledge gained from searching the information (continuous education?) Searching skills | Skills of pharmacist |
| 1. Continues education   Knowledge gained from continuous education (training, newsletter, emails)  /Motivation | Update his knowledge/ Self-motivated to improve |
|  | Pharmacist want to read more if there is new cases or complex cases |
|  | Motivation from boss or company/ workshop to update knowledge of the pharmacist |
|  | Program for updating and education |
|  | Updating knowledge of pharmacist by emails |
|  | Take time to update and learn but with a good impact on pharmacy practice |
|  | Updating from company or ministry of health better than self-learning |
|  | The company bears the cost of travel, the study of pharmacist |
|  | Updating of evidence or guideline |
|  | Sharing cases with the pharmacist |
|  | Motivation from boss or company/ workshop to update knowledge of the pharmacist |
|  | Updating knowledge of pharmacist by emails |
|  | Take time to update and learn but with a good impact on pharmacy practice |
|  | Updating from company or ministry of health better than self-learning |
|  | The company bears the cost of travel, the study of pharmacist |
|  | Updating of evidence or guideline |
|  | Sharing cases with the pharmacist |
| 1. Availability of evidence | Availability of evidence in pharmacy |
|  | Follow guideline |
| 1. Awareness campaigns   A need for awareness campaigns regarding pharmacist’s role in providing enough data to patients about medications | Interact with pharmacist role and identity (professional identity role) |
|  | Educate patient/public about medication by brochures |
|  | The conflict between guideline and experience |
|  | Convinced pharmacists |
| 1. Patient’ awareness/education /understanding /attitude and culture | According to patient-level of education |
|  | According to patient attitude |
|  | Patient culture/ education/ awareness |
|  | Patient culture and education |
|  | Low understanding/ education = barrier  High understanding/education = facilitator |
|  | Social media can hinder EB prescribing by spreading false or incomplete information about medications |
|  | Awareness campaigns increase the patient level of knowledge and understanding |
|  | F=high education level  H=low education level |
|  | low understanding about the role of the pharmacist |
|  | Patient awareness |
|  | Patient awareness /education |
|  | Patient understanding/education/awareness |
|  | Patient understanding/awareness/  Education |
|  | Patient understanding |
|  | Patient manner |
|  | Increase awareness of the patient/ Have awareness signs inside the pharmacy |
|  | Patients socioeconomic status |
|  | Patient comfort or satisfaction |
| 1. Patients counseling & pharmacist identity   Patients counseling empower pharmacist professional identity | Difficulty when prescribing OTC according to EB |
| 1. Is a difficult behavior   Pharmacist perceived prescribing OTC according to OTC is a difficult behavior | Convince them that the drug is not right for them |
|  | The difficulty of doing it without experience |
|  | Updating of evidence or guideline |
|  | Circumstances, difficulty |
|  | The willingness of pharmacists to adopt this innovation (diffusion of innovation theory) |
| 1. Putting constructive effort   Pharmacists are willing to put constructive effort to implement the behavior of OTC prescribing according to EBM | Diffusion of innovation theory |
|  |  |
| 1. Patients-pharmacists relationship   Established patients-pharmacists relationship through counseling, time and good communication skills facilitated OTC Rx according to EBM | Communication with patient |
|  | Counseling |
|  | Caregiver come to the pharmacy instead of the patient or missing information from the caregiver |
|  | Caregiver come to the pharmacy instead of the patient / missing information from caregiver /shyness |
|  | Discussion between patient and pharmacist |
|  | Skills, behavior, and communication of pharmacist with patients |
|  | Trust, convince for the patient /communication skills |
|  | Relationship, calls to the patient |
|  | Communication, take a history from the patient |
|  | Communication with patient and time spent with the patient |
|  | Miss communication |
|  | More communication faster and more EB prescribing |
|  | Communication skill of pharmacist |
|  | Active listening and attention when prescribing OTC more EB prescribing |
|  | Recurrence of the patient to the pharmacy and they have a good relationship |
|  | Trust between patient and pharmacist |
|  | No shyness or ego |
|  | Communicate with the doctor |
|  | Take time |
|  | Health care educator in pharmacy to overcome the time limitation |
|  | F, if time management  B, if time not managed correctly |
|  | The effect of pharmacy location on accordance with the physician location and other pharmacies |
|  | A need for electronic health care solutions to retrieve patients’ medical history and drug information |
|  | The pharmacist is an easy-access resource |
|  | Location of pharmacy/Near hospital = close support from the healthcare team |
|  |  |
| 1. Influence and power of other   influence and power of another pharmacist behavior on the in OTC RX pharmacist |  |
| 1. Availability and quality of pharmacist-physician channels of communication | Computer program give interaction of medication |
| 1. Limited time   The effect of the limited time available on pharmacist-patients communication | The computer program (health system) |
|  | Health system, computer or mobile program |
| 1. Electronic health care solutions   A need for electronic health care solutions to retrieve patients’ medical history and drug information | Computer program health system |
| 1. Pharmacy location   The effect of pharmacy location on accordance with the physician location and other pharmacies |  |
| 1. The pharmacist is an easy-access resource 2. Lack of OTC evidence-based treatment clear guidelines |  |
| 1. The pharmacist is an easy-access resource 2. Lack of OTC evidence-based treatment clear guidelines 3. Activate adhering to the current rules of dispensing and the need to determine the penalty for non-compliant | Availability of pharmacist, more time to contact him about medication |
|  | Barrier: availability of the drug  Facilitator: will give right drug even if not available |
| 1. Pharmacy management and higher administration | Availability of medication to all ages and condition |
|  | Availability of medication regardless of circumstances |
| 1. Activate adhering to the current rules of dispensing and the need to determine the penalty for non-compliant 2. Pharmacy management and higher administration 3. The need for checklists for prescribing make pharmacist follow the guideline and adhere to the policy 4. Adhere to generic name dispensing 5. The impact of health insurance | Penalty for anyone who doesn’t use EB and evaluation from company by mystery |
|  | Rules and government policies, and penalty/award from the government |
|  | Put with rules tools to apply and adhere to these rules |
|  | Generalization of rules to all pharmacy from the ministry of health for example/ government policies |
|  | Rules from the ministry of health |
|  | Announcing and clarification about guideline |
|  | Government policy to have some type of evidence in the pharmacy |
|  | when considering priority number one is the patient |
|  | Effect of repetition of cases  Repetition of the same disease states (practice) helps them remember the current EB  I think this is hinder because he will not keep updated with the new EB |
|  | Standard (WHAM) method for prescribing OTC specific for this pharmacy |
|  | Standard method (checklist) for prescribing OTC specific for this pharmacy |
|  | F, the generalization of checklist to all pharmacy  F, updating pharmacist about medication from FDA or ministry of health |
|  | Standard method (check list& WHAM) for prescribing OTC specific for this pharmacy |
|  | Brand name of medication/patient expectation |
|  | According to patient psychology, understanding, favor a particular brand name |
|  | Pharmacy management or. Support from boss |
|  | Management overlooks EB prescribing and just wants to sell |
| 1. Boss/Manager impact on the pharmacist | non-obligate pharmacist for target patient per day |
| 1. Pharmacy workflow management | Category manager should be the pharmacist |
|  | No target for patient or prescription per day |
|  | At the end of the day, they have a sales quota they must make, so they will give a non-EB drug just to make the sale, or have a loyal customer and make sure the patient is happy and will return |
| 1. The effect of job stress on pharmacist prescribing | Sales ignored for their patient ‘s benefit |
| 1. Effect of sales on prescribing | Evaluation from the ministry of health about pharmacist performance |
|  | Follow up/ Some sort of censorship or monitoring officials |
| 1. The effect of pharmacist’s evaluation | Complete evaluation from the company does not restrict sales |
|  |  |
| Modern and herbal medicine   1. The conflict between modern and herbal medicine on OTC R according to EBM | Competition to encourage pharmacist to prescribe depend on EB  Training for pharmacists when and how prescribing OTC medication?  Incentives from government or boss  Development, on the job training |
| 1. Incentives to dispense OTC according to EBM | Incentives, money and ticket flight |
|  | training help the pharmacist to prescribe OTC drugs according to checklist not sales  Put competition to encourage pharmacist to prescribe depend on EB |
|  |  |
| 1. The need for reminders for the pharmacist to prescribe according to EB |  |
| 1. Boring process |  |
| 1. Fears of patient reaction | No shyness or ego |
| 1. Lack of self-confidence (?) |  |
| 1. Pharmacist self-confidence, honesty, conscience |  |
